# An Integrated Molecular and Conventional Breeding Scheme for Enhancing Genetic Gain in Maize in Africa

**DOI:** 10.3389/fpls.2019.01430

**Published:** 2019-11-06

**Authors:** Melaku Gedil, Abebe Menkir

**Affiliations:** ^1^Bioscience Center and Maize Improvement Program, International Institute of Tropical Agriculture, Ibadan, Nigeria; ^2^Maize Improvement Program, International Institute of Tropical Agriculture, Ibadan, Nigeria

**Keywords:** maize, hybrid, molecular breeding, drought, *Striga*, genetic gain, West and Central Africa

## Abstract

Maize production in West and Central Africa (WCA) is constrained by a wide range of interacting stresses that keep productivity below potential yields. Among the many problems afflicting maize production in WCA, drought, foliar diseases, and parasitic weeds are the most critical. Several decades of efforts devoted to the genetic improvement of maize have resulted in remarkable genetic gain, leading to increased yields of maize on farmers’ fields. The revolution unfolding in the areas of genomics, bioinformatics, and phenomics is generating innovative tools, resources, and technologies for transforming crop breeding programs. It is envisaged that such tools will be integrated within maize breeding programs, thereby advancing these programs and addressing current and future challenges. Accordingly, the maize improvement program within International Institute of Tropical Agriculture (IITA) is undergoing a process of modernization through the introduction of innovative tools and new schemes that are expected to enhance genetic gains and impact on smallholder farmers in the region. Genomic tools enable genetic dissections of complex traits and promote an understanding of the physiological basis of key agronomic and nutritional quality traits. Marker-aided selection and genome-wide selection schemes are being implemented to accelerate genetic gain relating to yield, resilience, and nutritional quality. Therefore, strategies that effectively combine genotypic information with data from field phenotyping and laboratory-based analysis are currently being optimized. Molecular breeding, guided by methodically defined product profiles tailored to different agroecological zones and conditions of climate change, supported by state-of-the-art decision-making tools, is pivotal for the advancement of modern, genomics-aided maize improvement programs. Accelerated genetic gain, in turn, catalyzes a faster variety replacement rate. It is critical to forge and strengthen partnerships for enhancing the impacts of breeding products on farmers’ livelihood. IITA has well-established channels for delivering its research products/technologies to partner organizations for further testing, multiplication, and dissemination across various countries within the subregion. Capacity building of national agricultural research system (NARS) will facilitate the smooth transfer of technologies and best practices from IITA and its partners.

## Introduction

Maize (*Zea mays* L.) is a dominant food crop, occupying 40% of the total area planted with cereals in sub-Saharan Africa (SSA). It is high yielding, easy to process, and readily digested, and it costs less than other cereals. Maize grains, leaves, stalks, tassels, and cobs can all be used to produce a variety of food and non-food products. Its rich genetic diversity enables its cultivation across a range of agroecological zones in SSA. However, these zones are subject to high incidences of diseases, insect pests, and parasitic plants as well as drought, heat, and low soil fertility. Consequently, maize breeding activities conducted at International Institute of Tropical Agriculture (IITA) have been geared to tackling major production constraints that limit agronomic performance, increasing yield potentials and ensuring good post-harvest quality, improving nutritional quality, and reducing toxic substances. IITA researchers have used a wide range of conventional methods for breeding maize, including intra- and interpopulation improvement schemes, inbreeding and hybridization, and backcross selection to improve quantitative traits, such as yield potential, resistance to *Striga*, nitrogen use efficiency, tolerance to drought, resistance to stem borers, countering aflatoxin accumulation as well as nutritional quality. These breeding efforts have led to the generation and supply of diverse maize varieties, parental inbred lines, and hybrids of different maturity groups, grain colors, and types that evidenced both high yield potential and resistance to major diseases that are prevalent in the humid forests and moist and dry savannas of West and Central Africa (WCA). Such diseases include maize streak virus disease, southern corn leaf rust, southern corn leaf blight, *Curvularia* leaf spot, *Striga*, and downy mildew. These new maize varieties, which are also drought tolerant and demonstrate nitrogen use efficiency (NUE), account for 67% of all new maize cultivars released in the WCA region, contributing to increased productivity (Alene et al., 2009).

In spite of the significant progress of maize breeding to date, continued investments in this area to enhance productivity, agronomic fitness, and adaptation to climate change continue to be critical for sustaining agricultural growth and food security, improving nutritional quality, and securing harvests. In light of the remarkable advances in genomic tools, researchers at IITA, who are engaged in both molecular and conventional breeding, are collaborating with researchers at the International Maize and Wheat Improvement Center (CIMMYT) and at various universities and advanced research institutes in an effort to accelerate the breeding process through the application of DNA markers, genomics, proteomics, and laboratory-based assays for assessing resistance mechanisms and nutrient bioavailability. These cutting-edge scientific tools are of particular importance given the complex nature of the inheritance of drought tolerance, the efficiency of nutrient use, and resistance to diseases, insect pests, as well as parasitic plants in maize. Our aim, here, is to provide a review of the historical and current status of the application of genomic tools in maize breeding at IITA along with a road map for facilitating the integration of new tools and techniques that have the potential to accelerate genetic gain (Menkir et al., 2014; Abate et al., 2017; Atlin et al., 2017; Badu-Apraku and Fakorede 2018).

IITA’s maize improvement program (MIP), which is aimed at supplying farmers in WCA with productive and broadly adapted germplasm using conventional breeding methods, has achieved remarkable genetic gain over the past several decades, leading to increased maize yields in farmers’ fields (Menkir et al., 2012; Badu-Apraku et al., 2018; Menkir and Meseka 2019). Focusing on factors that limit agronomic performances, which include resistance to pests, diseases, and parasitic weeds as well as drought and soil fertility problems, researchers within the MIP have developed a number of distinct inbred lines, varieties, and hybrids for each agroecological zone. Impact studies, conducted within multiple countries in WCA at different times, have documented benefits of increased maize production associated with the improved varieties (Manyong et al., 2000; Alene et al., 2009; Wossen et al., 2017). Despite decades of work on breeding, there remains a considerable yield gap for maize in SSA (Nepolean et al., 2018). Reducing this gap, which can be attributed to several intertwined factors, is a complex challenge that calls for a multipronged solution that includes genetic improvement, optimal agronomic management, and enhanced agricultural inputs. The lofty goal of achieving sustainable agricultural productivity for staple food crops such as maize demonstrably benefits from advanced genomics-driven technologies and the rapidly evolving digital revolution. The history of maize breeding at IITA spans nearly five decades and has generated improved open pollinated varieties (OPVs) and hybrids. The diverse agroecological production zones, each with its unique biotic or abiotic constraints, are accounted for in the cultivar development process. These breeding strategies have generated maize varieties and hybrids with high yield potential and resistance to major diseases that are prevalent in humid forests and moist savannas as well as in mid-altitude areas in WCA. These diseases include lowland rust (caused by *Puccinia polysora*), lowland blight (caused by *Biopolaris maydis*), highland rust (caused by *Puccinia polysora*), highland blight (caused by *Exserohilum turcicum*), and gray leaf spot (caused by *Cercospora zea-maydis*). Newer threats, such as maize lethal necrosis (MLN) and fall army worm (FAW), have resulted in significant reductions in maize yields in many African countries. Moreover, a backdrop of climate change and deteriorating natural resources portend the emergence of new threats. To meet the needs of each maize-growing environment and its stresses in an effective way requires a prudently designed multidisciplinary strategy for developing suitable varieties for each of these environments. This review presents the current status of the application of molecular markers in the improvement process and attempts to formulate a road map that will enable the integration of new tools and techniques that can potentially accelerate genetic gain.

### Defining Product Profiles Based on Priority Traits

The MIP researchers are working to introduce critical revisions to existing cultivar development practices. The aim of these ongoing initiatives is to strengthen IITA’s crop breeding pipeline at all stages of the breeding process. These initiatives include developing rapid and effective characterization of germplasm, exploiting genetic diversity, adopting state-of-the-art data management systems, and applying modern statistical genomics approaches for genomic prediction. To facilitate the integration of these tools, the product profiles within the MIP have been revised, targeting the following agroecological zones: Sudan savannas, moist savannas, forest/transitional zones, and mid-altitude zones (**Table 1**). The target products/cultivars for each agroecology zone comprise different maturity classes (extra-early, early, medium, and late) as well as required basic trait, such as resistance to diseases or high yield potential, that are specific to each agroecological zone. Given the co-occurrence of different stresses within fields where maize is cultivated, IITA’s breeding program focuses on developing inbred lines, hybrids, and open-pollinated varieties that can tolerate multiple stresses. The new tools will help to accelerate the rate of genetic gains for such complex traits and enhance quality and other end-user preferred traits within product pipelines (**Table 1**).

**Table 1:** Current product profiles within International Institute of Tropical Agriculture (IITA’s) maize improvement program (MIP) categorized by maturity groups and agroecological zones.

Product Profile	Required traits									Desirable traits		
	Grain Yield	Resistance to *Striga*	Resistance to Southern Leaf Rust	Resistance to Southern Leaf Blight	Resistance to Maize Streak Virus	Resistance to *Curvularia* leaf spot	Resistance to *Tursicum* leaf blight	Resistance to Gray leaf spot	Resistance to Common rust	Pro-vitamin A content	Aflatoxin resistance	Nitrogen use efficiency (NUE)
**Extra-early** maturing maize varieties and hybrids adapted to the Sudan-savannas	X	X	X	X	X							x
**Early** maturing maize varieties and hybrids adapted to the Sudan-savannas	X	X	X	X	X							x
**Medium** maturing maize varieties and hybrids adapted to the moist-savannas	X	X	X	X	X	X					x	x
**Medium** maturing maize varieties and hybrids adapted to the forest/transitional zone	X		X	X	X	X			X		x	
**Late** maturing maize varieties and hybrids adapted to moist-savannas	X	X	X	X	X	X					x	x
**Late** maturing maize varieties and hybrids adapted to the forest/transitional zone	X		X	X	X	X			X		x	
**Late** maturing maize varieties and hybrids adapted to the mid-altitude zone	X				X		X	X	X	x		

The moist savannas are prioritized within the breeding program because they have the greatest potential for maize production. Major objectives for breeding maize varieties that are cultivated in this zone include traits of resistance to *Striga* spp., efficient use of soil nitrogen, drought tolerance, and early maturity. In the forest zone, prioritized traits are resistance to stem borers, foliar diseases, and ear rots. Maize varieties and hybrids cultivated at mid-altitudes must be resistant to the specific foliar diseases and ear rots that occur within this zone to manifest their high yield potential. Post-harvest quality is also an important concern that influences the adoption of improved varieties. Therefore, breeding materials are screened for dry milling quality and micronutrient content, and a collaborative initiative is underway to breed maize that is resistant to aflatoxin contamination. Key value-added traits that the MIP researchers are currently working on include carotenoid or pro-vitamin A (PVA) carotenoid content (Menkir et al., 2008b; Menkir et al., 2015b), mineral content of grains, for example, zinc and iron (Oikeh et al., 2004; Menkir 2008; Garcia-Oliveira et al., 2018), resistance to aflatoxin (Williams et al., 2015; Brown et al., 2016), and development of quality protein maize (QPM), which contains twice the quantity of essential amino acids lysine and tryptophan in the normal maize (Badu-Apraku et al., 2016b).

An example from the Sudan savanna zone of WCA, where growing cycles are short, illustrates how the productivity of maize can be enhanced for smallholder farmers. An appropriate cultivar must be high yielding at an extra-early stage (requiring 80–85 days to maturity) or early stage (90–95 days to maturity). In addition, it should be drought- and heat-tolerant and resistant to *Striga hermonthica* and foliar diseases. Value-added (desirable) traits could include resistance to aflatoxin and NUE. In the Guinea savanna, southern Guinea savanna, and forest/transitional zones, medium maturity (105–110 days) and late maturity (110–130 days) germplasm with the abovementioned traits would be appropriate. Germplasm developed for the forest/transitional zones should be resistant to ear rots and insect pests. In general, in all of the agroecological zones of WCA, newly developed cultivars must have the following traits: drought tolerance and heat tolerance, *Striga* resistance, and resistance to foliar diseases, such as southern corn rust, southern corn leaf blight, *Curvularia* leaf spot, and maize streak virus. Evidently, numerous traits need to be improved, requiring an efficient and effective breeding strategy for the timely delivery of products.

## Application of Molecular Tools to Accelerate Genetic Gain

**Figure 1:**
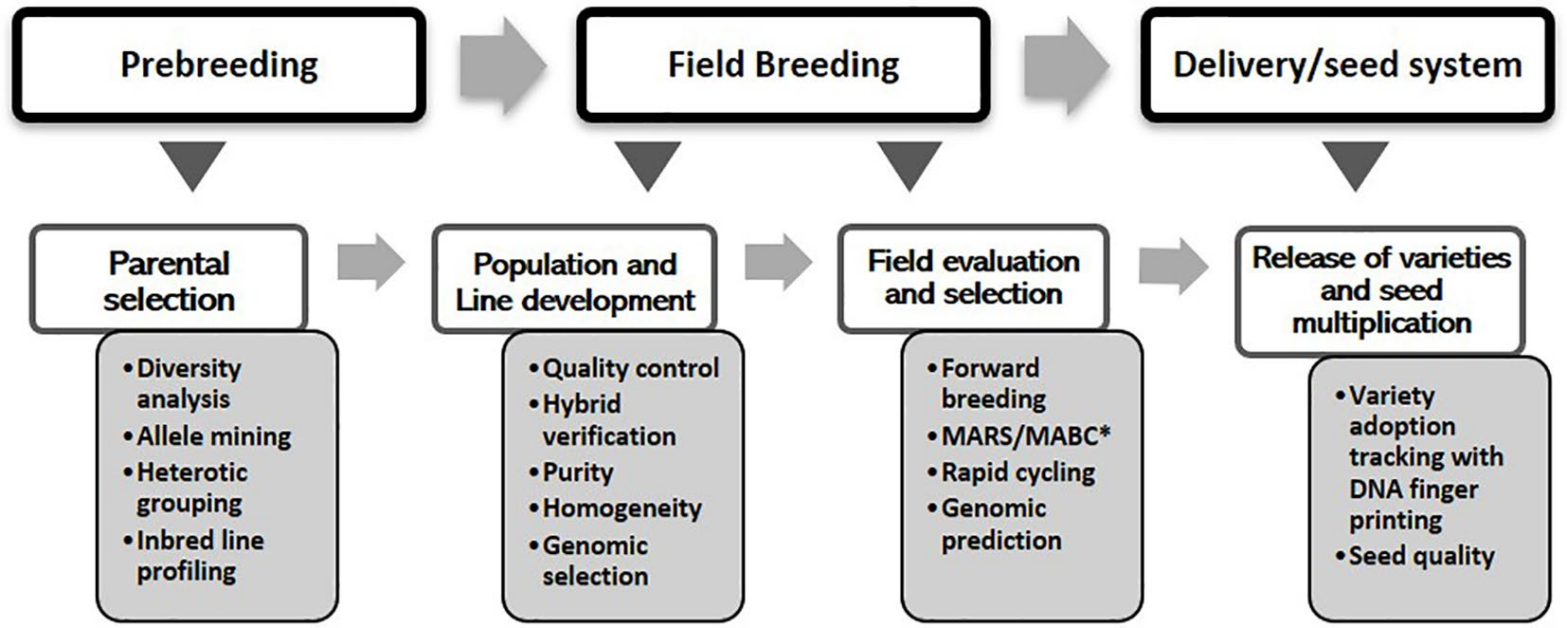
Genomics interventions within the maize cultivar development pipeline. *MARS, marker-assisted recurrent selection; *MABC, marker-assisted backcrossing.

The overall objective of the MIP is to exploit diverse germplasm to develop inbred lines, high-yielding varieties, and hybrids that can thrive under stresses occurring in different agroecological zones. The program’s cultivar development pipeline comprises pre-breeding, field breeding, and the delivery (final variety release and technology dissemination) stages. Under each stage, molecular tools were used for different purposes as shown in **Figure 1**. The maize breeding scheme at IITA can be broadly divided into categories of population improvement procedures that may lead to the development of OPVs or inbred line development for producing superior hybrids (Badu-Apraku and Fakorede 2018). A wide range of methods, entailing subtle variations, have been used both for population improvement and line development. For instance, population improvement methods could be for intrapopulations (e.g., mass selection or family selection) or for interpopulation improvement, which entails a variety of recurrent selection approaches. However, the first step in all breeding schemes entails choosing appropriate germplasm for constituting the population and germplasm pool. There are many sources of variation that maize breeders can consider. Currently, modern breeding tools, fueled by advances in genomics, and decision support systems, are emerging that can be used to harness these sources effectively.

Genomics interventions begin with the pre-breeding stage by tapping into the extensive genetic variations that exist within landraces and exotic germplasm. Within the MIP, the most widespread application of markers during the pre-breeding stage involves genetic characterization and diversity analysis to define heterotic groups and clusters that help planning of crosses for population development. Genomic tools provide an efficient method for accessing genetic variations in landraces for introgression into adapted germplasm (Gorjanc et al., 2016). The rapid evolution of next-generation genotyping and the concurrent development of informatics and data management tools have opened up unprecedented opportunities for advancing modern breeding systems. The emergence and rapid expansion of these ultra-high-throughput techniques have led to a dramatic reduction in the cost of sequencing, thereby creating an opportunity to evaluate all breeding lines and germplasm accessions. This development has enabled the deployment of genotype-based selection/prediction to enhance the productivity, nutritional quality, and resilience of food crops, including maize, leading to the generation and application of a wide variety of breeding schemes (viz. field breeding stage in **Figure 1**). To complement these fast turnaround and cost-effective genotyping platforms, high-throughput and precision phenotyping techniques are being developed to achieve increased genetic gain and rapid cultivar development. Substantial quantities of genotypic, phenotypic, and meta-data are required for this task, necessitating a system that can efficiently capture, store, manage, and integrate such big data. Moreover, there is a need for innovative methods of capturing and managing the high-volume data generated from molecular breeding to enable efficient data analysis and interpretation. Cutting-edge information and communication technology and robust statistical analyses are thus crucial for integrating all facets of conventional and genomics-assisted breeding.

Genomics is a field of paramount importance for understanding the genetic architecture of complex quantitative traits and characterizing germplasm collections to achieve accurate and precise manipulation of desirable alleles/genes. Apart from pursuing new discoveries, researchers within the MIP are applying molecular markers at various stages of the breeding process, including germplasm characterization, monitoring of line purity and genetic identity, parentage analysis, mapping of genes that control important traits, and population improvement through recurrent selection. An overview of their activities entailing the application of molecular markers to germplasm enhancement, improvement of grain quality, developing resistance to biotic and abiotic stresses, and quality control and tracking of varieties is presented in the following sections.

### Germplasm Enhancement

#### Diversity Analysis and Grouping

It is widely acknowledged that in the absence of sufficient knowledge of the extent of diversity within germplasm and the relationships among elite germplasm, significant advances in genetic improvement will be difficult to achieve. Therefore, the classification of inbreds into heterotic groups to maximize their potential usefulness in the development of productive hybrids and synthetics constitutes an important initial step in a breeding program. Breeders often perform parental selection for crosses using general combining ability (GCA), specific combining ability (SCA), and other methods. With the advent of molecular markers, genetic diversity analysis has been applied to group genotypes into heterotic groups and to select genetically distant parental lines for crosses. In the 1990s, markers such as RAPD, AFLP, and SSR were frequently used to analyze genetic diversity and to group genotypes based on degrees of genetic relatedness. With the emergence of high-throughput genotyping platforms such as genotyping by sequencing (GBS), the popularity of other types of markers has dwindled. The MIP research team is working with a diverse germplasm pool comprising different maturity groups, white and yellow grains, and adaptive trait donors derived from wild relatives such as Z. *diploperenis*. The team is using different methodological approaches, including testcross performance as well as phenological traits and pedigree information to assign inbred lines to different potentially complementary groups. The strategy adopted within the program is, on the one hand, to develop hybrids combining key traits. For instance, *Striga* tolerance is combined with drought tolerance (Mengesha et al., 2017), drought tolerance with heat tolerance (Meseka et al., 2018), or drought tolerance with low soil nitrogen tolerance (Ajala et al., 2019; Badu-Apraku and Akinwale 2011). Assessments of the genetic diversity of 128 lines that can tolerate drought and *Striga* that were conducted using single nucleotide polymorphism (SNP) markers have enabled the identification of suitable inbred lines for hybrid combinations based on genetic diversity (Mengesha et al., 2017). On the other hand, the integration of pedigree information with combining ability and genotypic analysis allows for more accurate definitions of heterotic groups (Badu-Apraku et al., 2016b). To complement the established method of grouping inbred lines according to the phenotypic values of genetic traits, which is essential for developing heterotic populations and synthetic varieties, markers are routinely used for defining heterotic groups in early and extra-early maturity groups (see Badu-Apraku and Fakorede 2018) or in medium to late maturity groups (Adebayo et al., 2015; Menkir et al., 2015a). The findings of a comparative study of various methods used to classify inbred lines into heterotic groups revealed that SNP-based genetic distance (GD) methods were effective for categorizing inbred lines into heterotic groups that were similar to groups formed using GCA- and SCA-based methods (Badu-Apraku et al., 2015a; Badu-Apraku et al., 2016a).

Molecular markers have also been effectively utilized in recurrent selection and composite population characterization. The application of an effective breeding scheme to identify and use divergent inbred lines is vital for the development of hybrids with elevated hybrid expression. One of the approaches used to develop inbred lines with good potential for generating superior hybrids is the use of reciprocal composites. An evaluation of 36 test crosses formed from S_4_ lines extracted from the third reciprocal recurrent selection (RRS) cycle generated the best hybrids that yielded up to 50% more grain yield than commonly grown commercial hybrids in Nigeria (Menkir et al., 2015a). In this study, marker-aided diversity analysis was found to be a useful tool for efficiently developing improved reciprocal composites that could serve as sources of new and divergent parents for developing productive hybrids, in addition to the technique of introgressing novel alleles for broadening and diversifying the genetic base of adapted germplasm. In a similar study, the extent of genetic diversity and changes in allele frequency of two composites derived from four cycles of reciprocal recurrent selection were assessed using SNP markers (Kolawole et al., 2017). Studies such as those cited above confirm the usefulness of molecular analysis in broadening the genetic base of populations, thereby fostering sustained genetic gain in long-term breeding strategies.

In the case of parental selection for high carotenoid accumulation, the first step entailed an assessment of 38 orange and yellow endosperm maize inbred lines for their genetic diversity. Parental lines with varying carotenoid content were drawn from the two distinct groups of orange and yellow endosperm maize formed by AFLP markers (Adeyemo et al., 2011). The hybrids formed by crossing lines from different groups were tested in multiple environments, which led to the identification of several hybrids with the traits of high PVA content and desirable agronomic performance (Menkir et al., 2014). Furthermore, one of the applications of molecular markers is the classification of early maturing maize lines in heterotic groups. The grouping of early white QPM lines using the SNP-based method was compared with that using GCA-based methods and found to be more efficient (Badu-Apraku et al., 2015a; Badu-Apraku et al., 2015b).

#### Identification and Exploitation of Diverse Sources of Germplasm for Genetic Enhancement

The use of exotic germplasm for strengthening economic traits such as improved yields is steadily rising. The successful incorporation of exotic germplasm into tropical germplasm has been limited because of the poor adaptation of this germplasm to tropical climates and to exposure to new diseases and insects. Nevertheless, there are several examples of the effective utilization of exotic germplasm to maximize heterosis in hybrids. Various germplasm development strategies have been used within the MIP to broaden the gene pool of maize. For example, DNA-based genetic characterization and diversity analyses have proved to be valuable methods and tools for decision making for maximizing the utilization of the main classes of genetic resources, namely wild relatives of crops, landraces, and improved elite lines (Menkir et al., 2006; Menkir et al., 2007). Rare novel traits can be introgressed from wild relatives more effectively through marker-assisted backcrossing within a relatively shorter time period compared to conventional backcrossing methods. For example, tropical germplasm with the traits of nutrient-rich grain (Menkir et al., 2015b) and resistance to parasitic weeds (Menkir et al., 2005: Amusan et al., 2008) was successfully developed through the introgression of wild relatives and exotic germplasm.

### Improvement of Nutritional Quality and Secure Harvests

#### Biofortification With Pro-Vitamin A

The development of fast, inexpensive, and flexible genotyping methods that can be deployed quickly along with accurate plant phenotyping facilities is imperative for the mainstreaming of genomic-assisted breeding. Accordingly, we have selected priority traits of economic importance for genomics intervention. The prevalence of micronutrient deficiencies in the diets of millions of people in developing countries has prompted mega-initiatives such as HarvestPlus (https://www.harvestplus.org/). IITA’s partnership with HarvestPlus is aimed at enriching maize with three critical micronutrients, of which vitamin A (VA) is a priority, with limited work conducted on iron and zinc. The evaluation of large amounts of genetic material is necessary to develop maize cultivars biofortified with VA. Given the high cost and low turnaround time of high-performance liquid chromatography-based quantification of carotenoids, and the narrow genetic base of carotenoids within tropical maize endosperm, the use of predictive markers is unquestionably the best alternative for enhancing genetic gain in the PVA content of maize. Marker-assisted selection (MAS) for PVA in maize is considered an appropriate target in the area of molecular breeding of crops such as maize. In light of the discovery of key genes involved in the carotenoid biosynthesis pathway, variations in nucleotides that influence the accumulation of carotenoids in maize endosperm have been categorized in temperate maize germplasm (Harjes et al., 2008; Yan et al., 2010). The effect of these functional gene markers on the accumulation of PVA in tropical maize has been investigated at IITA (Azmach et al., 2013), in which polymerase chain reaction (PCR)-based DNA markers developed from three key genes—PSY1, *lcyE*, and *crtRB1*—were assessed for their effectiveness in marker-assisted selection for PVA content in 130 tropical maize lines. Whereas the PSY1 markers were found to be monomorphic for favorable alleles across all inbred lines, two of the three markers from *crtRB1* (5’TE and 3’TE) were found to be strongly associated with carotenoid accumulation. Furthermore, these two functional markers evidenced linkage disequilibrium, indicating the possibility of including either one in MAS. Genotypes featuring the two markers produced up to three times more carotenoid than genotypes that lacked the favorable alleles. *lcyE* was mainly associated with lutein but not with PVA within these sets of inbred lines. However, genotypes with a combination of the favorable alleles, *crtRB1* and *lcyE*, showed better PVA accumulation than genotypes carrying the individual genes. Although these markers are predominantly predictive, some inconsistencies arose in this study as well as in previous studies (Vignesh et al., 2012), as some lines carrying unfavorable alleles of *crtRB1* or *lcyE* expressed high levels of carotenoid. Such inconsistencies in the effects of the predictive markers indicate the need for further refinement and optimization of the markers using diverse sources of germplasm.

Subsequently, these same 130 lines, with germplasm of mixed genetic backgrounds derived from tropical and temperate origins developed at IITA, were further investigated for genome-wide associations using GBS to search for additional genes (Azmach et al., 2018). The analysis revealed several significant association signals, most of which were colocalized with the known carotenoid biosynthesis genes, namely *crtRB1* (chromosome 10), *lcyE* (chromosome 8), and *ZEP* (chromosome 2). The findings of the study predictably confirmed the significant effects of the two major (known) carotenoid biosynthesis genes: *lcyE* and *crtRB1*. In addition, significant novel associations were detected for several transcription factors and uncharacterized protein coding genes. The effect of the markers may have been confounded by other, uncharacterized carotenoid biosynthetic pathway genes whose genetic effects have not yet been evaluated. This explanation is corroborated by the results of the genome-wide association study (GWAS) (Azmach et al., 2018).

Gebremeskel et al. (2018) also conducted marker-trait association to confirm the efficacy of the markers on another set of 108 inbred lines and to validate the effect of the *Zep1* gene. The findings of the study confirmed the significant effect and association of *crtRB1* markers (5’TE and 3’TE) with carotenoid accumulation. However, they revealed that Zep-SNP(801) only affected the accumulation of α-carotene. The overall conclusion that can be drawn from these studies is that a higher number of favorable alleles within a genotype is associated with a higher quantity of total PVA content. Therefore, marker-assisted selection conducted for haplotypes with favorable alleles of the three genes that are located on three separate chromosomes is expected to result in a substantial improvement in PVA content. In addition to developing and testing a new marker associated with carotenoid content, the MIP team have also developed an in-house option of using an optimized protocol for genotyping SNP markers. This alternative protocol, known as Tetra-ARMS PCR, can be implemented easily and quickly (Medrano and de Oliveira 2014). Moreover, the process of fine-tuning prediction accuracy and optimizing the carotenoid markers is ongoing. In collaboration with advanced labs located in the United States, IITA researchers are working to identify additional functional markers that contribute to carotenoid accumulation.

In addition to VA, iron and zinc are also recognized as critical micronutrients that can be augmented through breeding (Garcia-Oliveira et al., 2018). Because breeding iron- or zinc-fortified tropical adapted maize is of lower priority compared with PAV-fortified maize, there is limited information available on genetic variations of tropical maize relating to these two minerals. However, the findings of an evaluation of hundreds of low-land and mid-altitude maize inbred lines are encouraging, revealing the existence of sufficient genetic variation relating to iron and zinc (Menkir 2008). Beginning with the first stage of identifying markers for efficient selection, efforts to develop tropical maize enriched with these two important minerals can generate a new product profile with a further value-added trait.

#### Resistance to Aflatoxin

The contamination of maize produce with mycotoxins produced by fungi that cause ear rot in maize compromises food quality and poses a potential health hazard in Africa. IITA and its partners have made laudable efforts to minimize mycotoxin contamination in maize by developing and deploying integrated pre- and post-harvest management practices that mainly include biological control in which nontoxigenic strains are used to outcompete toxigenic strains (Udomkun et al., 2017). In addition, these partners have collaboratively developed maize cultivars that are resistant to fungi that produce aflatoxin and fumonisins fungi (Menkir et al., 2008a). Extensive studies have been conducted on the regulation of aflatoxin biosynthesis. Moreover, omics-based investigations focusing on the mechanism of resistance to aflatoxin in crops have led to the identification of several genes and signal molecules. However, further investigations are needed to develop a fuller understanding and to identify targets for the development of molecular tools for the efficient breeding of aflatoxin-resistant maize varieties (Fountain et al., 2015). Although there are still significant knowledge gaps relating to the biochemistry of maize x *A. flavus* interaction, quantitative trait loci (QTL) analyses conducted with SSR or SNP markers for various types of populations have revealed putative QTLs with variable phenotypic variance explained (PVE) (Warburton and Williams 2014). More consistent QTL regions have been identified through the confirmation of QTLs from a different genetic background and through a multi-environment evaluation (Willcox et al., 2013). Moreover, using association mapping, Warburton et al. (2013) identified a new source of resistance to *A. flavus.* IITA researchers have used SNP markers linked to these QTLs to develop SNP marker assays for deployment in MAS/Marker-assisted backcrossing (MABC) for introgressing aflatoxin resistance to elite lines.

### Breeding for Resistance to Biological Threats

#### Maize Streak Virus

Among the many biotic factors responsible for heavy losses in maize yields in WCA, the most important viral diseases and pests in the region are the maize streak virus (MSV), parasitic weeds (*Striga hermonthica*), and stem borer insects (*Sesamia calamistis* and *Eldana saccharina*). These biotic issues are priority research areas in the MIP. Discovery work, including association analysis and QTL analysis using bi-parental mapping populations, is currently in progress for three biotic issues at IITA. Breeding to develop resistance to MSV disease (MSVD) has historically been one of the top priorities of the MIP and nearly four decades ago, it was considered a basic component for maize research in Africa (Efron et al., 1989; Fajemisin 2014). The identification of first-generation molecular markers provided a boost for the conventional backcross-based method to convert susceptible introduced germplasm to resistant varieties. A major QTL accounting for up to 76% of phenotypic variation was identified in CIMMYT-developed inbred lines and fine mapped to an interval of 0.87 cM on chromosome 1. SNP markers associated with this major QTL have been developed and used in forward selection (Nair et al., 2015). Resistance to MSVD is still heavily reliant on a single major locus of resistance (*msv1*). The MIP team began searching for additional genes conditioning resistance to the disease. The use of QTL analysis in bi-parental mapping populations resulted in the identification of additional QTLs (Ladejobi et al., 2018), pointing to additional QTLs on a different chromosome. In addition, a different population was used to identify new loci underlying MSV resistance in maize using 93 F_2:3_ families. In this ongoing study, putative QTLs accounting for considerable phenotypic variation were colocalized and considered to be of particular interest because these QTLs had similar locations where clusters of genes influence resistance to viral diseases in maize, including MSVD. Following further dissection and refinement, the QTL identified in this study may emerge as a powerful tool for marker-assisted selection to improve MSV resistance in maize that could help breeders to deal with a possible outbreak of a new strain of MSVD in Africa.

#### Striga

*Striga* is a noxious parasitic weed that poses a serious threat to maize production in the savannas where the weed is endemic. Maize being a new introduction in the savanna, host plant resistance does not exist in landraces and requires introgression from exotic germplasm. Problems relating to *Striga* are further exacerbated by deteriorating soil fertility, the recurrence of drought, and the expanding and changing cropping systems that are conducive to heavy infestation with *Striga*. *S. hermonthica* is the most widespread species, causing colossal economic damage to maize and other cereals. Research on host plant resistance to *Striga* dates back to the 1980s (Fajemisin 2014; Menkir and Meseka 2019). Tolerance of *Striga* is a complex trait that is strongly influenced by genotype x environment interaction. Conventional breeding utilizes elite tropical advanced lines, African landraces, as well as wild maize relatives, such as the teosinte, *Zea diploperennis*, to identify sources of resistance to *Striga*.

A multi-pronged approach has been adopted to identify mechanisms enabling the resistant maize cultivar, ZD04, to tolerate *Striga* infestation and to determine the genetic control of resistance to *Striga*. This strategy encompasses QTL analysis in bi-parental crosses, association analysis, differential expression analysis as well as the use of biochemical, physiological, and morphological assays. Ongoing studies at IITA were designed to complement IITA’s existing studies. These include several ongoing collaborative projects: one with the University of Sheffield in the United Kingdom, focusing on both pre- and post-attachment, a second joint study with Purdue University that compares the mechanisms underlying differences between a resistant line containing genes derived from a wild species of maize and a susceptible tropical line, and a third collaboration with the University of Florida. This last study focuses on introgressing a transposon-induced mutant, *zmccd8*, which has very low levels of *Striga* germination stimulants, into IITA’s elite breeding lines.

#### Stem Borers

Among the many pests that attack maize, stem borers, particularly the pink stem borer (*Sesamia calamistis*) and the sugarcane borer (*Eldana saccharina*), wreak extensive damage. To dissect the genetic bases of resistance to the abovementioned stem borers, an F2 maize population with 238 lines was developed. Leaf material was collected from this population, and its DNA was genotyped using GBS technology. A map was developed for this F_2_ population. The F_2:3_ population was planted in Ibadan, Nigeria, in 2018. The results of preliminary QTL analyses of the 238 F3 lines genotyped using SNP markers revealed several genomic regions on several chromosomes that affect stem borer resistance traits, namely leaf damage, tunnel length, and leaf feeding at the terminal stage. The majority of the genomic regions identified in this ongoing study coincided with those found in previous studies conducted on resistance to various borers and FAWs. The same F3 population is being cultivated in Ibadan to conduct further phenotyping and to verify the results obtained using data from the first year. Increasing the throughput and accuracy of phenotyping techniques is expected to generate sufficient phenotypic data for performing robust marker-trait association analysis.

### Breeding for Drought and Heat Tolerance

Given limited and erratic rainfall in the savanna regions of WCA, drought tolerance is an essential trait for developing a resilient and targeted maize cultivar for this region. In this agroecological zone, drought commonly occurs simultaneously with other stresses, including heat, low nitrogen, and *Striga*. Conventional approaches have been used to improve the performance of maize hybrids under conditions of drought and heat (Meseka et al., 2014; Meseka et al., 2018) and for early maturing inbred lines facing drought and low nitrogen stress (Badu-Apraku and Akinwale 2011). These approaches led to the identification of promising hybrids and parents that produce up to 26% higher yields than the best commercial hybrid. However, repeatability was found to be low for yields under drought stress. The complexity of phenotyping for drought is further compounded by the simultaneous occurrence of other stresses. Furthermore, drought can happen at various stages of growth with varying effects, with stress encountered during the flowering stage being more severe than during other stages. Evaluating genotypes for drought tolerance is a daunting task because of annual fluctuations in weather conditions and a lack of uniformity of trial fields. The development of long-term facilities to help control environmental variations in the field (soil, water, and temperature) is therefore necessary. Furthermore, physiological characterization of drought-related traits facilitates an understanding of the mechanisms underlying drought tolerance. Facilities for conducting thorough phenotyping of reasonably large numbers of genotypes can generate invaluable phenotypic data for association with genotypes, which can be utilized in molecular breeding. To enable rapid advances in genetic enhancement for fostering abiotic stress resilience, it is imperative to develop robust, large-scale screening facilities (Cobb et al., 2013).

### Quality Control

Marker-based quality control (QC) is essential for ensuring purity and true-to-type maize genetic material within maize breeding programs. Mainstreaming QC using DNA markers provides breeders with rapid and cost-effective tests of the homozygosity of inbred lines, the homogeneity of populations, and the fidelity of crosses. Attempts to utilize markers for QC have been initiated in the era of low-throughput SSR marker assays (Warburton et al., 2010; Semagn et al., 2012). The rapidly declining cost of SNP genotyping has opened up an opportunity for the routine use of SNP markers for QC. At IITA, both SSR and SNP in-house assays are used to verify crosses before selfing them for mapping population development. Furthermore, given extensive genetic variability within maize OPVs, it is necessary to analyze large and representative samples of individuals for each OPV, which is difficult, costly, and time-consuming. Genotyping with SSRs using pools of individuals from a population has proven to be much more efficient than characterizing several individual plants within each population (Warburton et al., 2010). Bulk samples of 8 to 15 individual plants were used in numerous studies conducted on genetic diversity with or without replication (Warburton et al., 2010) to represent inherent genetic diversity within populations. However, few studies have been conducted to assess whether bulk samples containing more than 30 plants per population would provide better classification of OPVs with molecular markers. Whereas the use of markers for QC in inbred lines and hybrids is straightforward, its application in OPV can be complicated.

IITA has generated a large number of improved maize OPVs with high yield potential, PVA accumulation, resistance to diseases and *Striga*, tolerance of drought, and low levels of aflatoxin production from diverse sources of germplasm (Menkir et al., 2012). Many improved OPVs have been registered and released for cultivation by resource-poor farmers in WCA. An understanding of the genetic relationships among these OPVs will prove useful prior to their distribution to farmers to minimize the potential risk associated with genetic uniformity under variable growing conditions. A study was conducted to compare bulk sample sizes of 30, 50, and 100 plants, aimed at ascertaining an optimal sample size for accurate and cost-effective DNA fingerprinting of open-pollinated varieties of maize and hybrids through genotyping by sequencing (Arafayne et al., 2018). In this study, a sample size of 30 plants was found to be optimal with respect to cost effectiveness and efficient characterization of heterogeneous and heterozygous maize populations and OPVs. Further optimization and protocol development are needed for analyzing hybrids, parents of hybrids, and other types of genetic materials.

### Adoption Tracking

Tracking and identifying crop varieties using DNA-based genotyping is gaining increasing acceptance as an approach for determining rates of adoption of improved crop varieties by farmers. Recent studies of cassava (Rabbi et al., 2015; Floro et al., 2018), sweet potato (Kosmowski et al., 2018), and other crops have shown this to be a reliable method for assessing the distribution of improved varieties *vis-à-vis* landraces or local varieties preserved by farmers. DNA-based monitoring and tracking of farmer-grown seeds also revealed the ages of varieties, pointing to an urgent need for further work on variety turnover. This work requires access to a well- curated reference DNA fingerprint library of officially released and registered varieties against which unknown samples randomly collected from farmers’ fields can be compared. Subsequently, the farmers’ varieties (unknown samples collected during surveys) can be matched with specific library samples based on pairwise genetic similarities calculated using all of the markers. Genotyping markers of choice are those markers that exhibit SNP because of their genomic abundance, ease of scoring, and amenability to high-throughput genotyping using next-generation sequencing techniques.

## Strategies for Genomics-Enabled Breeding

### Improved Breeding Schemes

The introduction of affordable and high-throughput genotyping technologies has prompted the development of breeding schemes that incorporate indirect marker-based selection. Some of the commonly used strategies are MABC, which is used to introgress markers that are closely linked to major genes or to a few loci from unadapted donor parents to adapted recipient parents, thereby shortening the conventional backcrossing scheme. MABC is being used at IITA to introgress aflatoxin resistance from temperate donor lines to tropical lines. Where markers linked to major genes are not available, which is the case for most economically important complex traits, marker-assisted recurrent selection (MARS) has been used to improve populations by increasing the frequency of favorable alleles for the trait of interest (Beyene et al., 2016; Abdulmalik et al., 2017; Bankole et al., 2017).

#### Marker-Assisted Recurrent Selection

MARS has been applied to two populations within the MIP, with the aim of accumulating favorable alleles within a family of lines derived from bi-parental crossing. The first population, which was propagated through the crossing of two parents that combined tolerance to *Striga* and drought, was subjected to two cycles of genotypic selection in which the best parents were selected based on genotypic estimated breeding value and recombined to generate subsequent cycles (Abdulmalik et al., 2017; Bankole et al., 2017). In these initial experiments aimed at verifying theoretical concepts and performed under the Drought Tolerance Maize for Africa project, test crosses of 50–60 randomly selected S1 lines, extracted from each cycle, were evaluated within different environments with and without stresses. Evaluations of test crosses within multiple environments showed relative genetic gain in grain yield for at least one of the cycles. Marker-based assessments of changes in frequency of favorable alleles also showed increments of favorable alleles for advanced cycles, thereby demonstrating the effectiveness of MARS for improving grain yield without compromising other desirable traits.

Similar studies were conducted by CIMMYT in eastern and southern Africa under the abovementioned project. A slightly modified MARS technique, in which a subset of significant markers was used to estimate the breeding value of individual plants, was selected for assessment of 10 bi-parental maize populations (Beyene et al., 2016). Evaluations of test crosses of all 10 populations along with commercial checks under water-stressed and well-watered conditions revealed genetic gain (up to 16%) in grain yield under both drought and optimal environments. Beyene et al. (2015) also used a genomic selection model incorporating all available marker information (200–300 SNPs) to predict genetic values of individuals/progenies developed from eight bi-parental crosses. After three selections at 10% intensity had been conducted, all of the cycles of all of the populations were evaluated in test crosses grown in drought and optimal environments. The average genetic gain per cycle of 0.086 Mg ha^-1^ was recorded. Hybrids derived from the third selection cycle produced significantly higher yields than the base population (the zero cycle) as well as up to 7% yield gain compared with hybrids developed using the conventional pedigree method.

#### Forward Breeding

Currently, MAS is widely applied for traits controlled by major genes or quantitative trait loci in maize and other major crops. A high-throughput genotyping project (HTPG), supported by Bill and Melinda Gates Foundation (BMGF) and led by The International Crops Research Institute for the Semi-Arid Tropics (ICRISAT) (http://cegsb.icrisat.org/high-throughput-genotyping-project-htpg/; accessed on July 29, 2019), was established to support Consultative Group on International Agricultural Research (CGIAR‘s) breeding programs by creating a low-cost genotyping platform to facilitate the implementation of an accelerated crop improvement program. The aim of forward breeding is to increase the frequency of rare alleles by performing early generation screening for specific traits using highly informative, diagnostic, and preferably functional markers. The traits that will be initially considered are single/oligo gene traits as well as substantially large-effect QTLs. However, in cases where phenotyping approaches are cheaper as well as accurate and fast, MAS is not necessary. Prioritizing traits and evaluating the efficiency of the two approaches is a prerequisite for developing an efficient and effective breeding program. The initial genotyping service will focus on trait-linked and validated SNPs that have been converted to KASP assays. Although a large number of trait-linked markers are currently in use, there is still a lack of markers for key traits. Furthermore, for some traits, the accuracy of marker prediction needs to be improved. The continuous process of new marker development and validation is pivotal to achieve maximal benefits during and beyond the project. The adoption and integration of forward breeding within the existing breeding pipeline will require extensive changes in their design. At IITA, some level of integration of forward breeding is underway in the case of traits for which validated predicted markers are available, including markers for PV, MSV resistance, reduced aflatoxin, and QPM.

#### Genomic Selection and Prediction

Genomic selection (GS) has been proposed as alternative to widely deployed MAS strategies, such as MABC, for use with complex traits (Heffner et al., 2010; Xu et al., 2017). The peculiarity of GS is that it involves the use of all marker information for calculating genomic estimated breeding values (GEBVs), which can be used as an indirect selection criterion in the absence of further phenotyping, thereby accelerating genetic gain by shortening the breeding cycle. Rapid advances in molecular biology, resulting in affordable and high-throughput genotyping have paved the way for the application of GS in plant breeding (Meuwissen et al., 2001). In the GS scheme, an appropriately sized training population is genotyped and phenotyped, and the GEBV is estimated. Subsequently, the testing population is only genotyped, and the selection is made solely based on GEBV. An optimized GS approach requires, among other things, the relatedness of the training population to the test population. In general, two different GS approaches are being evaluated. Whereas GS is a method of rapid cycling that is used to predict additive effects (breeding value) in early generations (e.g. F_2:3_), genetic prediction (GP) focuses on predicting both additive and non-additive effects, thereby enabling the total genetic values of individuals to be estimated (Crossa et al., 2017).

Different GS models have been proposed for predicting genetic values, with the aim of accelerating genetic gain (Bernardo and Yu 2007; Crossa et al., 2014). For instance, prediction models based on individual marker effects and on haplotypes have been developed and tested in the area of crop improvement to enhance prediction accuracy (Jiang et al., 2018). In light of growing appreciation of the potential value of GS for accelerating genetic gain, a number of molecular breeding schemes that vary slightly have been developed and deployed within public sector maize improvement programs (Beyene et al., 2015; Edriss et al., 2017). However, their levels of prediction accuracy also vary and are influenced by several factors, including the heritability of the trait under investigation and the relationship between the training and test populations (; Jannink et al., 2010; Zhao et al., 2012; Crossa et al., 2017). Although the findings of preliminary studies are promising, further studies are needed to demystify prevailing genetic and statistical complexities and to integrate GS effectively within breeding pipelines for routine implementation. Recent succinct reviews have revealed varying perspectives on the use of genomic-enabled prediction in combination with high-throughput precision phenotyping and pedigree-based selection (Crossa et al., 2017; Makanza et al., 2018). A discussion of challenges that include statistical complexities and prediction accuracy entailed in the implementation of GS is beyond the scope of this review. However, the methods applied in GS and GP are the subject of extensive research, globally, on various crops and animals. It is anticipated that statistical models will continually evolve and be improved and refined through the incorporation of pedigree, genomic, and environmental data. IITA could accordingly utilize historical multi-environment phenotype data, accumulated over decades, for training GS models. Ongoing CGIAR initiatives aimed at establishing a cost-effective genotyping platform and the development of analytical tools/pipelines and continual open trainings on GS techniques are anticipated to stimulate the adoption of molecular breeding techniques within public sector research institutions in Africa.

#### Doubled Haploid

An affordable and efficient doubled haploid (DH) induction process is pivotal for the rapid development of fixed maize lines that can be used within the breeding pipeline or in genetic and genomic studies. The role of DH in enhancing breeding efficiency and accelerating genetic gain in maize has been discussed elsewhere (Prasanna et al., 2012). DH has been recognized by CRP-MAIZE (https://maize.org) of CGIAR as one of the modern and powerful tools that can enhance maize breeding efficiency. In light of the goal of breeding efficiency, CIMMYT has established a state-of-the-art DH facility at the KARLO-Kiboko Center in Kenya, which provides a DH line development service for the breeding programs of all African partner organizations, including CIMMYT, IITA, national agricultural research and extension system (NARES), and small and medium enterprises (SMEs) DH technology, including the development of tropically adapted inducer lines with improved haploid induction rates, has been successfully optimized within CIMMYT’s global maize program (Chaikam et al., 2018). Integration of DH-derived lines in the maize breeding pipeline has led to hybrid development (Yoseph et al., 2017). The use of DH in the MIP has, however, been limited mainly because of logistical and plant quarantine issues (fear of seed-based transmission of MLN from East Africa). However, efforts are underway to establish a DH facility in WCA to enhance the integration of DH technology in the breeding scheme.

### High-Throughput Phenotyping

In the current context of a proliferation of high-throughput genotyping options, the accuracy and throughput of phenotyping are factors limiting the rate of genotype–phenotype association, slowing down marker-assisting breeding. The main applications of high-throughput phenotyping (HTP) in molecular breeding are bi-parental mapping populations for linkage analysis, association panels comprising unrelated lines, or a GS training population. Many of the agronomically important traits are complex and require quantitative multi-environment evaluation methods. Accurate phenotyping of such complex traits entails precision and a reduction in environmental effects. Phenotyping is conducted within a controlled environment, such as a water-stressed or optimal environment, or in the case of diseases and pests, through artificial infection and infestation, respectively. Phenotyping of such intractable traits is not only expensive and cumbersome but also are very low throughput. For biotic stresses, the most conventional and affordable field phenotyping requires the use of hot spots, but the level of throughput lags well behind the genotyping throughput. For instance, whereas hundreds of samples can be genotyped within a few weeks, phenotyping of such large samples can take several months and requires extensive resources. Innovative phenotyping approaches, requiring the construction of high-tech tools and platforms, including remote sensing and imaging, are currently being developed (Fahlgren et al., 2015 Bai et al., 2016; Reynolds and Langridge 2016; Brichet et al., 2017; Araus and Kefauver 2018). In the short term, boosting existing hot spots for disease screening or identifying and securing suitable sites for long-term phenotyping of abiotic stresses, such as drought and heat, is essential for enhancing the generation of large volumes of phenotypic data at a particular time. Next, we offer specific recommendations for selected priority traits.

#### Maize Streak Virus

The phenotyping protocol for MSV depends on artificial infestation of the test lines and populations with a laboratory-reared viruliferous leafhopper colony under glasshouse conditions. Evaluation criteria for post-MSVD infection include MSV severity at various stages (4, 5, and 6 weeks after infection), percent recovery, and area under the disease progression curve. This procedure is cumbersome, time-consuming, and low throughput. Enhancing MSV phenotyping throughput is essential for discovery and validation studies such as GWAS and linkage mapping. It is essential to capitalize on the rapid advancement of biotechnologies to enhance the accuracy and speed of detection and the categorization of diverse pathogens, including MSV. Establishing a dedicated facility for disease screening and harnessing novel technologies, such as image processing methods, should be considered to improve speed, accuracy, and consistency (Singh and Misra 2017).

#### Striga Hermonthica

Given the evolving and diverse parasitism of *Striga* ecotypes, it is important to develop diverse genetic stocks to facilitate rapid cultivar development aimed at addressing emerging and future production challenges. In contexts where breeding involving artificial infestation is time-consuming and challenging because of the complex nature of the host–parasite relationship and its intersection with other environmental factors, the use of molecular marker technology is expected to increase the efficiency of breeding crops with complex traits such as resistance to *Striga*. An understanding of the specific mechanisms of resistance based on knowledge of host–parasite biology as well as identification of genomic regions linked to *Striga* resistance can provide the impetus for rapid germplasm development. Moreover, it can improve the efficiency of deliberate introgression of genes from diverse germplasm sources to cultivated maize varieties. Furthermore, the identification of different resistance mechanisms will be useful for pyramiding several resistance factors that may discourage the development of new races or ecotypes of *Striga*. Presently, screening for *Striga* resistance is a laborious task requiring the injection of ethylene gas into the soil to stimulate suicidal germination of existing *Striga* seeds in the field prior to artificial infestation. *Striga*-infested and non-infested plots are purposefully arranged across fields, with *Striga*-infested blocks forming strips that are back-to-back with non-infested blocks (Berner et al., 1997). However, employing such protocol to phenotypic evaluation of large population sizes for GWAS or bi-parental mapping population, due to low throughput and expensiveness and laboriousness, becomes impractical. The use of newly introduced stimulants such as sphynolactone-7 as a substitute for ethylene (Uraguchi et al., 2018) along with other modifications may improve the throughput, lower the cost, and enhance the efficiency and accuracy of the screening process.

#### Fall Army Worm

An invasion of *Spodoptera frugiperda* (J E Smith), commonly known as the fall army worm (FAW) was first reported in WCA (Goergen et al., 2016) and is the latest threat to have emerged after the MLN pandemic, causing economic damage to Africa’s maize production (Baudron et al., 2019). Host plant resistance to FAW constitutes a pivotal component of appropriate integrated pest management for African smallholder farming systems. Efforts to breed for FAW has been initiated by screening available advanced materials, since no Africa-adapted source of resistance is known until the arrival of FAW in Africa (Prasanna et al., 2018).

#### Maize Lethal Necrosis

Maize lethal necrosis (MLN), an aggressive and devastating viral disease that was first reported in Kenya in 2011 and quickly spread to neighboring countries, is one of the latest woes facing African agriculture (Mahuku et al., 2015; Redinbaugh and Stewart 2018). As a result of the rapid and concerted response of an international coalition to the MLN outbreak, aimed at preventing the transmission of MLN through seeds, the outbreak was contained within certain countries in Eastern Africa (https://mln.cimmyt.org/). However, despite the existence of strict regulations and quarantine measures, the spread of this disastrous pathogen to WCA is imminent (Isabirye and Rwomushana 2016), posing a potential risk to maize production within the region. Cognizant of the dire threat that MLN poses to food security, CIMMYT’s global maize program has launched an intensive breeding program (Beyene et al., 2017) that is supported by a dedicated screening platform and advanced genomics tools and techniques (https://mln.cimmyt.org/). IITA’s breeding programs include a forward breeding scheme aimed at preemptively introgressing MLN resistance into elite advanced lines.

#### Value-Added Traits

The nutritional quality of maize grain is an important component of the maize program, which includes routinely performed macro- and micronutrient analyses. IITA has a well-equipped food utilization and nutritional laboratory where various nutrients, including carotenoids, iron, zinc, and starch are analyzed. Occasionally, when large volumes of samples need to be analyzed to match the genotype populations, the option of outsourcing to external service providers is availed of. Another important desirable trait is aflatoxin resistance. The current protocol for phenotyping is adequate for the phenotyping needs of the program.

## Product Deployment and Delivery to Farmers

### Technology Transfer and Capacity Building of NARS

Enhancing the capacity of NARS partners in all aspects of maize breeding and product delivery is one of the key objectives of IITA and its partner institutions within CGIAR. Various approaches have been used to transfer technology, including the formation of a subregional network and multidisciplinary NARS projects encompassing multiple countries. Beginning from the late 1970s, the increasing demand and diffusion of improved maize products, particularly hybrids, to areas outside of the traditional maize-belt zones, such as the savanna regions, have prompted the establishment of regional projects such as Semi-Arid Food Grain Research Development, which later became the West and Central Africa Collaborative Maize Research Network. The latter project is engaged in regionally coordinating the development and promotion of maize germplasm (Badu-Apraku et al., 2011; Badu-Apraku and Fakorede 2018). The network, which has secure funding from USAID and other donors, has successfully facilitated regional alliances and capacity building of all stakeholders involved in the value chain that contribute to boosting maize productivity. Similarly, the Drought Tolerant Maize for Africa project launched in 2007, which was subsequently reconceived as the Stress Tolerant Maize for Africa project, galvanized a multidisciplinary team of scientists, development workers, and private sector partners, including CIMMYT, IITA, and NARS, from 13 countries in SSA (Abate 2015). Notable among the many achievements of the above project has been the deployment of genomics-aided breeding schemes and other tools that have enabled a reduction of the breeding cycle, enhancing breeding efficiency and contributing to the release of hundreds of varieties across the target countries (Abate 2015; Beyene et al., 2015; Beyene et al., 2016; Abdulmalik et al., 2017; Bankole et al., 2017).

### Variety Turnover

Even though nearly 500 maize varieties have been released in SSA, only a handful of older varieties (>30 years old) are grown over a vast area across multiple countries, according to a recent survey (Abate et al., 2017). Smallholder farmers in Africa who are engaged in seed businesses generally use or distribute varieties developed decades ago. There are apparent knowledge gaps relating to the numbers, ages, and identities of these varieties (Atlin et al., 2017). An Africa-wide survey conducted in 2013–2014 found that on average, 36% of the total cultivated area was planted with improved varieties (OPV or hybrid) (Abate et al., 2017). The average age of the varieties was 15 years, indicating much slower variety turnover in this region compared with the variety turnover in other regions, worldwide. While there are several possible explanations for farmers’ and seed producers’ choices of a popular older variety, it is striking that the measured parameters in the above survey showed considerable variation among regions, signifying the need for customized mitigation within each region. As IITA embarks on a modern breeding scheme, supported by genomics-enabled selection, HTP phenotyping, and digital data capture, ensuring the continuous replacement of existing varieties with improved ones will be essential to meet market demands and climate change-induced stresses.

### Impact Assessments

The impacts of maize research conducted over 25 years in WCA have been assessed on the basis of the performances of the different varieties and their adoption trends within multiple countries. Alene et al. (2009) found that nearly 50% of the new maize varieties released in 11 countries in WCA in the 1990s were sourced from IITA. The constant supply of stress-tolerant germplasm, coupled with improved crop management practices, has contributed to the significant expansion in maize production in many countries in WCA, notably Benin, Burkina Faso, Cameroon, Gambia, Ghana, Mali, Senegal, and Togo. Wossen et al. (2017) conducted a household-level survey in Nigeria, with the aim of evaluating the impacts of drought-tolerant maize varieties on productivity, poverty, and food security. Their analysis of the data revealed that adoption of these varieties contributes to productivity gains and poverty reduction, averting the risk of food scarcity among adopters. The use of DNA fingerprinting for reliable variety identification is invaluable in such impact studies.

## Prospects and Perspectives

The main factors driving efforts to modernize maize breeding programs in Africa include the need to accelerate the rate of genetic gain and variety turnover as well as the availability of new technologies and tools that are affordable and accessible (Xu et al., 2017). One of the strategies for achieving this goal is the introduction and customization or optimization of tried and tested modern tools, services, and best practices derived from the private sector, including improved operational efficiency. A major goal of the maize breeding program at IITA is to exploit diverse germplasm to develop improved populations, inbred lines, and hybrids that are adapted to lowland and mid-altitude ecologies in WCA. Ultimately, these improved germplasm that demonstrate resilience to the various biotic and abiotic constraints in this region will be transferred to NARS partners to accelerate their efforts to make good quality seeds available to farmers in WCA. Diverse conventional breeding methods have been applied, including intra- and interpopulation improvement schemes, inbreeding and hybridization, backcross selection, and the use of selection indices. In recent years, a number of suitable biotechnological tools and schemes have been incorporated within the various stages of the breeding pipeline, with the aim of accelerating genetic gain. Molecular markers have been used to characterize the patterns of genetic diversity of elite inbred lines and to separate them into heterotic groups as well as to identify QTLs linked to key quantitative traits.

The rapidly advancing field of molecular biology and associated technologies has produced a plethora of affordable “omic” resources, including high-quality reference genomes, transcriptomes, small-RNA profiles, methylome, proteome, and metabolome. Prominent among these resources are next-generation sequencing-based assays, which are having a profound effect on genotype–phenotype associations, enabling biological studies to be conducted at higher resolutions and larger scales. Consequently, multi-pronged genomics-based approaches are being employed to elucidate the roles of genetic factors in the expression of economically important agronomic traits. Their application ranges from germplasm enhancement through the characterization of genetic variation to accelerated cultivar development through efficient selection schemes.

A number of initiatives have been developed to enhance the efficiency and effectiveness of breeding programs through process optimization and modernization [e.g. Excellence in Breeding (EiB) http://excellenceinbreeding.org/]. Recruitment of state-of the-art technologies for developing varieties, effective trial management practices, and the mechanization and digital tools and devices are expected to enhance the rate of genetic gain in farmers’ fields (Cobb et al., 2019). The rapid cultivar development process will focus on enhancing crop productivity and increasing nutritional quality and quantity under optimal and suboptimal growth conditions.

**Figure 2:**
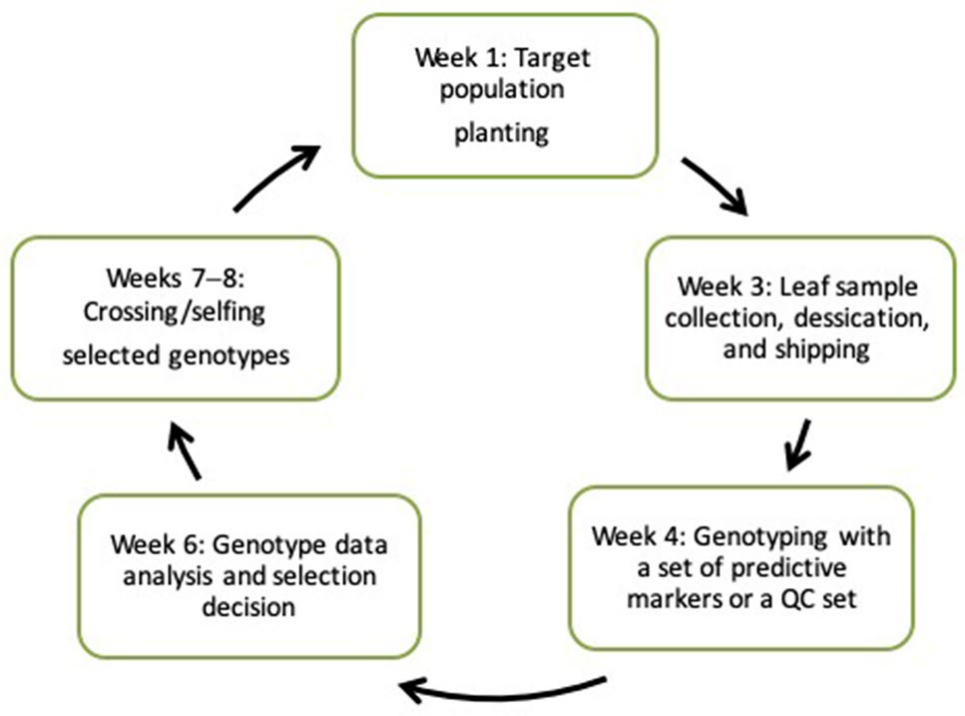
The seasonal forward selection cycle in maize breeding.

New technologies stemming from the development of next-generation “omic” technologies and the concomitant ICT revolution are now accessible to researchers engaged in maize improvement programs in developing countries, especially within Africa. The assorted tools, services, and knowledge that are accessible through these technologies provide a foundation for transforming breeding pipelines through rapid breeding cycles (Atlin et al., 2017). The changing landscape of genomics and the advent of high-throughput, cost-effective genotyping service providers have rendered molecular laboratories unnecessary. Recent CGIAR-wide initiatives, such as HTPG/EiB, have facilitated the provision of affordable, fast turnaround genotyping services for multiple crops. Under this arrangement, a researcher develops populations, mainly for forward selection, collects leaf tissues from seedlings, and ships them to the nearest service center. Genotype data are returned within 2 weeks, enabling selection decision making to occur prior to the flowering stage (**Figure 2**). Private service providers also offer genotyping of large samples at competitive prices.

The key elements of molecular breeding are high-throughput genotyping, high-throughput precision phenotyping (remote sensing, image analysis, etc.), digital data capture, and data management, which includes appropriate data analysis and decision making. The mechanization of trial management and other best practices also contribute to the efficiency of the breeding pipeline. Optimizing trial management and developing mechanized phenotyping facilities, entailing some level of automation and digitalization, are some of the measures that can be envisaged for achieving an efficient and effective breeding pipeline.

IITA’s breeding program is proactively fostering partnerships with CIMMYT and other advanced research institutions to boost its internal capacities, which will ultimately facilitate timely harnessing of emerging technological opportunities. Because IITA serves as the technology gateway to NARS partners, it should routinely use an optimized, state-of-the-art modern breeding system that can be easily adopted by partners through dissemination within established technology transfer pathways.

The steady advances in omics and related technologies warrant the adoption of new tools and methodologies. Therefore, breeding programs should be appraised and revised by incorporating new innovations. Furthermore, the efficiency and effectiveness of the breeding pipeline should be monitored by employing metrics designed to measure the impacts of breeding outcomes on the ultimate users, namely farmers.

## Conclusion

There are numerous agricultural constraints affecting WCA’s maize-growing region, including diseases, parasitic weeds, drought, heat, and low soil fertility. Farmers’ lack of access to quality seeds of improved maize varieties coupled with limited availability of agricultural inputs and nonuse of appropriate management practices are some of the factors that lead to low productivity. Genetic improvement of inbreds and hybrids can address the most common biotic and abiotic constraining factors. However, there are several factors, such as a lack of agricultural inputs (improved seeds, fertilizers, and herbicides), cultural practices (planting density and tillage), and socioeconomic factors that contribute to reduced maize yields and that cannot be addressed through genetic improvement alone. Nevertheless, developing an updated maize improvement strategy that is sustainable, underpinned by genomics and related technologies, will significantly enhance genetic gain of maize yields from farmers’ fields. A comprehensive improvement plan that encompasses upstream discovery-oriented research and pre-breeding along with actual field breeding and the deployment of breeding products through the galvanizing of all stakeholders, including the private sector, is essential for efficient seed delivery.

The main purpose of this review has been to provide an overview of the current status of the application of genomics and to provide perspectives on how breeding could be modernized by tapping into genomics-enabled innovative tools and techniques. Integrating molecular breeding with conventional breeding for accelerated genetic gain is now feasible within public sector research programs in developing countries. Globally, the multidisciplinary research community is actively working on various approaches for developing optimized schemes that enhance genetic gain. Distilling through innovative applications and adopting suitable breeding strategies is critical. Such strategies should be sufficiently flexible to leverage new genomics-based technologies to halt the onslaught of endemic and emerging threats such as MLN and FAW that are wreaking devastating losses in maize production in Africa. In an era of increasing access to genomic resources, the importance of eliminating the phenotyping bottleneck cannot be overemphasized. Upgrading and expanding phenotyping platforms to promote resilience to heat and drought, pests, and diseases, and *Striga* should be prioritized in WCA.

Given the drastic reduction in the cost of marker assays, the use of DNA-related technologies to achieve accurate varietal identification is gaining wide acceptance. At IITA, DNA markers are being used to assess seed purity and varietal identities. This technique, when combined with socioeconomic studies, can be used in assessments of the adoption of different varieties.

In general, public sector maize breeding programs in developing countries are attempting to tackle a host of agricultural constraints that include existing as well as emerging threats. This demands constant prioritization to ensure efficient use of the meager research facilities and human resources that are available. Nevertheless, researchers in developing countries can draw on the vast genetic diversity at their disposal and on diverse screening and testing sites. The rapid advance of new breeding technologies in recent years, propelled by the omics revolution, can potentially accelerate genetic gain for high-priority adaptive traits as well as value-added quality traits. Nevertheless, careful assessment is necessary prior to the adoption of new technologies. Insightful reviews of the challenges entailed in the application of molecular markers in plant breeding have revealed the various issues associated with marker-trait association through QTL analysis and have led to proposals of methods for the efficient application of markers in the absence of QTL mapping (Bernardo 2008). The advent of biotechnology has triggered the development of a wide array of tools and techniques aimed at improving the effectiveness and efficiency of breeding. At the same time, certain techniques that have been widely promoted and deployed have shown limited success (Bernardo 2016). Stakeholders associated with agricultural research communities (donors, policy makers, and development workers) tend to promote particular techniques (e.g., GWAS or GS), considered as the most effective solution for achieving rapid gains in cultivar development. However, numerous publications have revealed the limited effectiveness of marker-aided selection for complex economic traits such as yield and drought tolerance. Various approaches, including physiological breeding, which combines refined phenotyping protocols for physiological traits, have been proposed in combination with GS and remote sensing (Reynolds and Langridge 2016). Drawing on lessons learned over the past several decades, researchers at IITA are carefully assessing and empirically testing the usefulness of particular technologies for achieving their goals.

A final point relates to the stepwise introduction of technologies such as genome editing, doubled haploid, remote sensing, and imaging systems that can potentially revolutionize breeding programs. Furthermore, speed breeding has emerged as a novel technique for shortening generational cycles (Watson et al., 2018). Although establishing such facilities in developing country may not be foreseeable in the near future, the conventional off-season generation advancement method could be expanded to accommodate larger populations. The integration of these innovative techniques with genome selection and HTP phenotyping will undoubtedly lead to accelerated genetic gain.

## Author Contributions

MG conceived the review topic, drafted the manuscript, and designed the figures. AM contributed to the write-up and reviewed the final draft.

## Funding

This work was supported by the CGIAR Research Program on Maize (MAIZE) and by the Bill & Melinda Gates Foundation and USAID funded project titled “Stress Tolerant Maize for Africa” (grant number OPP1134248), The MAIZE program receives financial support from the governments of Australia, Belgium, Canada, China, France, India, Japan, Korea, Mexico, the Netherlands, New Zealand, Norway, Sweden, Switzerland, the U.K., and the United States, and from the World Bank.

## Conflict of Interest

The authors declare that the research was conducted in the absence of any commercial or financial relationships that could be construed as a potential conflict of interest.
